# Initially elevated arterial lactate as an independent predictor of poor outcomes in severe acute pancreatitis

**DOI:** 10.1186/s12876-020-01268-1

**Published:** 2020-04-19

**Authors:** Wenqing Shu, Jianhua Wan, Jie Chen, Wenhua He, Yong Zhu, Hao Zeng, Pi Liu, Yin Zhu, Liang Xia, Nonghua Lu

**Affiliations:** Department of Gastroenterology, The First Affiliated Hospital of Nanchang University, 17 Yongwaizheng Street, Nanchang, Jiangxi 330006 PR China

**Keywords:** Arterial lactate, Poor outcomes, Severe acute pancreatitis

## Abstract

**Background:**

The present study aimed to investigate the relationships between arterial lactate levels and outcomes in severe acute pancreatitis.

**Methods:**

The study retrospectively analyzed the medical data of 329 patients with severe acute pancreatitis from January 2014 to February 2019. We compared baseline characteristics, laboratory data, severity scores, types of persistent organ failure, and primary and secondary outcomes of patients with and without elevated arterial lactate levels at admission. A multivariate logistic regression analysis model and receiver operating characteristic curve were adopted to evaluate the value of arterial lactate ≥4 mmol/L for identifying high-risk patients. Trends in arterial lactate levels were compared between patients in the survivor and nonsurvivor groups over a period of 7 days.

**Results:**

Compared to normal arterial lactate levels, patients with elevated arterial lactate levels show significantly higher incidences of multiple persistent organ failure (3% vs 30%, *P* < 0.01), death (2% vs 11%, *P* < 0.01), septic shock (4% vs 24%, *P* < 0.01), pancreatic infection (12% vs 37%, *P* < 0.01), abdominal compartment syndrome (3% vs 20%, *P* < 0.01), pancreatic necrosis (41% vs 63%, *P* < 0.01), and a need for ventilator support (26% vs 54%, *P* < 0.01). For predicting mortality, arterial lactate levels ≥4 mmol/L had a high hazard ratio (10, 95% CI; 3.7–27; *P* < 0.01) and the highest area under the curve (0.78).

**Conclusions:**

Our results indicate that initially elevated arterial lactate is independently associated with poor outcomes and death in patients with severe acute pancreatitis and may serve as an early high-risk stratification indicator.

## Background

Severe acute pancreatitis (SAP) is one of the most dangerous acute inflammatory diseases in the abdomen, with the characteristics of rapid progression, serious complications and high mortality. As the most lethal classification of acute pancreatitis (AP), the 15–20% of AP patients may develop into SAP and mortality in SAP patients may be as high as 30–50% [1–3]. Considering the morbidity and mortality associated with SAP, the ability to identify high-risk patients in the early stages of the disease is critical because it can help clinicians institute more effective management or quickly transfer the patient to specialist care to improve the clinical prognosis.

The methods of risk stratification and prediction of severity in the early period of AP have been developed over the decades, including several clinical scoring systems and laboratory parameters, such as the Acute Physiology and Chronic Health Examination (APACHE II) [4, 5], systemic inflammatory response Syndrome (SIRS) [6, 7], blood urea nitrogen (BUN) [8–10], creatinine [11], hematocrit [10], serum calcium [9], procalcitonin [12], C-reactive protein (CRP) [13] and base excess (BE) [9, 14]. However, as the most serious classification of AP, there are few articles concerning the poor outcomes associated with SAP. In addition, all of the above methods are complicated or lack high accuracy [15] and are therefore of restricted clinical use and are not recommended by the new Atlanta international consensus and American guidelines [1, 2]. Thus, it is necessary to find a new marker or a simple scoring system to identify high-risk patients with SAP.

Lactate is a product of anaerobic metabolism of glucose and is generally considered to be a marker of tissue hypoxia. In addition, previous studies have also found that the presence of elevated arterial lactate levels reflects critical tissue hypoperfusion, which is strongly associated with increased morbidity and mortality in critically ill patients [16–21]. A recent study has shown that elevated serum lactate is a new biomarker that could be an important tool in predicting poor outcomes of AP on admission, especially in predicting death [22]. Thus, we assume that arterial lactate may be a potentially promising biomarker to risk-stratify patients with SAP. However, the association between arterial lactate levels and outcomes in SAP has not been reported to date.

Therefore, we designed a retrospective study with the aim of exploring the relationships between the levels of arterial lactate and outcomes in SAP. Furthermore, we intended to record the average arterial lactate value over 7 days between survivor and nonsurvivor groups in patients with SAP.

## Methods

### Study design and patient population

The present study was a retrospective analysis of adult patients diagnosed with SAP from January 2014 to February 2019 at the First Affiliated Hospital of Nanchang University. The review boards of The First Affiliated Hospital of Nanchang University center approved the study protocol (No. 2011001).

**Fig. 1:**
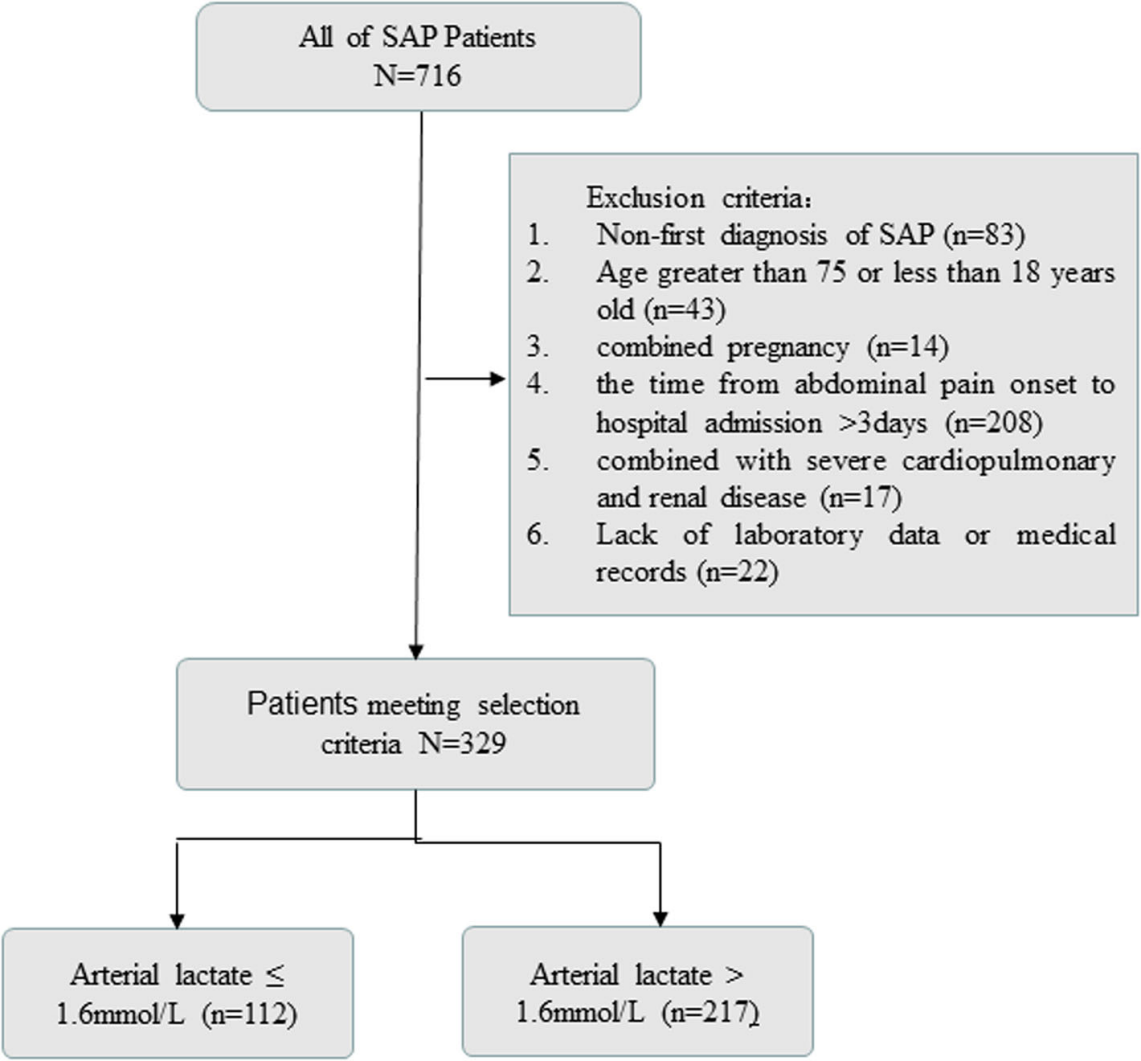
Selection process and results (arterial lactate strata) for severe acute pancreatitis

Considering the early identification of high-risk patients with SAP, patients enrolled must meet the following criteria: first episode of SAP; age less than 75 or greater than 18 years old; time from abdominal pain onset to hospital admission of less than 3 days; patient’s information recorded in the database; the first arterial lactate tested within 2 h after hospitalization; and other biomarkers measured within 24 h. The exclusion criteria were the following: SAP during pregnancy; patients with severe cardiopulmonary and renal disease before the development of SAP; lack of laboratory data or medical records. Based on their initial lactate measurement, patients were divided into normal and elevated groups. The specific selection process is shown in Fig. 1.

### Definitions

The diagnosis of AP was based on the presence of at least two of the three following criteria [1]: characteristic abdominal pain [2]; elevation in serum amylase and/or lipase greater than three times the upper limit of normal; and [3] characteristic imaging findings of AP by abdominal computed tomography scan [1]. The patient was classified as SAP based on the presence of persistent organ failure (POF) for more than 48 h (defined by modified Marshall scoring system≥2). Organ failure included the cardiovascular, and/or pulmonary, and/or renal systems, accompanied by a systolic blood pressure < 90 mmHg after continuous fluid resuscitation, PaO_2_ / FiO_2_ ≤ 300 or the need for mechanical ventilation, serum creatinine level > 1.9 mg/dl after rehydration or hemodialysis without preexisting renal disease [1]. The patient was classified as having a pancreatic infection based on the presence of gas collections in the pancreatic and/or peripancreatic tissues on contrast-enhanced computed tomography or based on a positive Gram stain and culture for bacteria and/or fungi from a fine-needle aspiration biopsy, percutaneous catheter drainage, and endoscopic transluminal drainage [1]. The diagnosis of septic shock was based on Chinese guidelines for management of severe sepsis and septic shock in 2014, meeting the following criteria: hypotension caused by sepsis (systolic blood pressure < 90 mmHg or drops more than 40 mmHg) after fluid therapy [23]. The definition abdominal compartment syndrome (ACS) base on the World Society of the Abdominal Compartment Syndrome. Intra-abdominal pressure. IAP measured by Indirect monitoring urinary bladder pressure via Foley bladder catheter. ACS is defined as a sustained IAP > 20 mmHg that is associated with new organ dysfunction [24].

### Outcomes

The primary outcomes in this study included death within 1 month of follow-up, septic shock, and peripancreatic infection. The secondary outcomes included abdominal compartment syndrome, pancreatic necrosis, walled-off necrosis, portal vein thrombosis, pancreatic fistula, intestinal fistula, diabetes, the rate of ventilator support, hospital stay and hospital stay in the intensive care unit (ICU). Telephone and outpatient follow-up were arranged for a month following discharge.

### Statistical analysis

Statistical analysis was performed using IBM SPSS software. Continuous data are presented as the means and interquartile range and analyzed by Student’s t-test and the Mann-Whitney U test. We used the Chi-square test or Fisher exact test to analyze categorical variables and show them as numbers (frequencies). Some indicators have been further analyzed with uni- and multivariate logistic regression analyses performed with a Cox regression model and reported as the hazard ratio (HR) and 95% confidence intervals (95% CIs). The arterial lactate level was measured continuously for 7 days. Some patients could have multiple measurements in 1 day, in which case the highest value was used. Compared with the previous day, the recorded arterial lactate value on the next day must be separated by 6 h. ANOVA test used to analyze multiple measurements of lactate. *P* value ≤0.05 was considered statistically significant.

## Results

### Baseline characteristics between patients in the normal group and the elevated group

A total of 716 patients were diagnosed with SAP, of whom 329 met the criteria for inclusion in this study (Fig. 1). Table 1 shows the patient demographics, laboratory data and several clinical scoring systems at admission of the overall SAP population and study cohort. There were 112 SAP patients with normal arterial lactate levels and 217 SAP cases with elevated levels. The average age of the normal group was 51.8 years, and 59% of the patients were male. The mean age of the elevated group was 51.2 years, and 59% of the patients were male. There was not statistically significant difference between the normal and elevated groups in demographics (consisting of age, sex, etiology, body-mass index), partial laboratory data (such as white blood count, serum calcium, serum albumin C-reactive protein), the SIRS scores and the Balthazar grading standard. Compared with the normal group (*N* = 112), patients in the elevated group (*N* = 207) had significant differences in hematocrit (41 (36.6–46.1) vs 44.4 (40–50), *P* < 0.01), serum glucose (7.7 (6.1–9.7) vs 10.5 (8.0–14.7), *P* < 0.01), serum urea (5.7 (4.1–9.6) vs 7.7 (5.6–12.4), *P* < 0.01), serum creatinine ≥1.9 mg/dl (10% vs 30%, *P* < 0.01), procalcitonin ≥3.8 ng/mL (21% vs 55%, *P* < 0.01), absolute value of base excess ≥4 mmol/L (27% vs 65%, *P* < 0.01) and APACHE II scores ≥15 (12% vs 25% *P* < 0.01).

**Table 1:** baseline characteristics in the 329 subjects by arterial lactate stratum

Variable	All patients (*n* = 329)	Normal lactate (*n* = 112)	Elevated lactate (*n* = 217)	*P* value
Age, years, (SD)	51.4 ± 13.8	51.8 ± 13.2	51.2 ± 14.2	0.71
Sex (male), n (%)	194 (59%)	66 (59%)	128 (59%)	0.74
Etiology, n (%)				0.06
Biliary	161 (49%)	65 (58%)	96 (44%)	
Alcoholic	42 (13%)	15 (13%)	27 (12%)	
Hyperlipidemia	102 (31%)	27 (24%)	75 (35%)	
Other	24 (7%)	5 (5%)	19 (9%)	
Body-mass index, Kg/m^2^, (SD)	24.6 ± 3.7	24.2 ± 3.7	24.5 ± 3.7	0.82
Balthazar grading standard, n (%)				0.13
C- grade	26 (8%)	13 (12%)	13 (6%)	
D- grade	222 (67%)	76 (68%)	146 (67%)	
E- grade	81 (25%)	23 (20%)	58 (27%)	
Laboratory data				
White blood count, × 10^9^/L, (SD)	14.3 ± 6.8	15.2 ± 5.4	14.9 ± 6.3	0.23
Hematocrit, IQR	43.1 (38.9–48.9)	41 (36.6–46.1)	44.4 (40–50)	< 0.01
Hematocrit ≥44, n (%)	154 (47%)	35 (31%)	119 (59%)	< 0.01
Serum calcium, mmol/L,	1.9 (1.7–2.1)	2 (1.8–2.2)	1.9 (1.6–2.1)	0.01
Serum calcium< 2 mmol/L, n (%)	182 (55%)	50 (45%)	132 (61%)	0.06
Serum glucose, mmol/L, IQR	9.3 (7.3–12.7)	7.7 (6.1–9.7)	10.5 (8.0–14.7)	< 0.01
Serum urea, mmol/L, IQR	7.3 (5.0–11.5)	5.7 (4.1–9.6)	7.7 (5.6–12.4)	< 0.01
Serum creatinine≥1.9 mg / dl, n (%)	74 (22%)	10 (9%)	64 (29%)	< 0.01
Serum albumin	35 ± 5.4	35.4 ± 5.3	34.8 ± 5.4	0.37
C-reactive protein ≥150 mg/L, n (%)	269 (82%)	90 (80%)	179 (82%)	0.64
Procalcitonin≥3.8 ng/mL, n (%)	142 (43%)	23 (21%)	119 (55%)	< 0.01
BE ≥|4|mmol/L, n (%)	170 (52%)	30 (27%)	140 (65%)	< 0.01
Severity scores				
SIRS scores ≥3, n (%)	128 (39%)	36 (32%)	92 (42%)	0.07
APACHEIIscores, IQR	10 (7–13)	9 (7–12)	11 (8–14)	< 0.01
APACHEIIscores≥15, n (%)	68 (18%)	13 (12%)	55 (25%)	< 0.01

Data are mean (SD), median (IQR), or n (%); *SD* Standard deviation, *IQR* Interquartile range, *BE* Base excess, *SIRS* Systemic inflammatory response syndrome, *APACHE* Acute Physiology and Chronic Health Evaluation

### Comparison of types of POF in a week after admission between the normal and elevated groups

Considering that the main cause of early death in SAP is POF [25], we compared the relationship between lactate levels and the types of POF in a week after admission. Table 2 demonstrates that all of 104 (93%) patients developed a single POF, and the respiratory system was involved in 99 (88%) patients in the normal group, which is far from the situation in the elevated group, in which 151 (70%) patients developing a single POF, and the respiratory system was involved in 139 (64%) patients. However, multiple POF was present in 8 (7%) patients (7 lung and kidney and 1 lung, kidney and heart), which was obviously below the 66 (20%) patients suffering multiple POF in the elevated group (43 lung and kidney, 3 lung and heart, and 18 of lung, kidney and heart). Compared to patients with normal arterial lactate levels, we observed that patients with high arterial lactate have significantly higher incidences of developing multiple POF (7% vs. 20%; *P* < 0.01).

**Table 2:** types of persistent organ failure after 48 h at admission and different groups by arterial lactate

Variable	All patients (*n* = 329)	Normal lactate (*n* = 112)	Elevated lactate (*n* = 217)	*P* value
Single persistent organ failure				
Respiratory	238 (72%)	99 (88%)	139 (64%)	< 0.01
Renal	17 (5%)	5 (5%)	12 (6%)	0. 8
Cardiovascular	0 (0%)	0 (0%)	0 (0%)	1
Multiple persistent organ failure				
Respiratory + renal	52 (16%)	7 (6%)	45 (21%)	0.01
Respiratory + cardiovascular	3 (1%)	0 (0%)	3 (1%)	1
Respiratory + cardiovascular + renal	19 (6%)	1 (1%)	18 (8%)	0.01

### Comparison of primary and secondary outcomes according to different groups

**Fig. 2:**
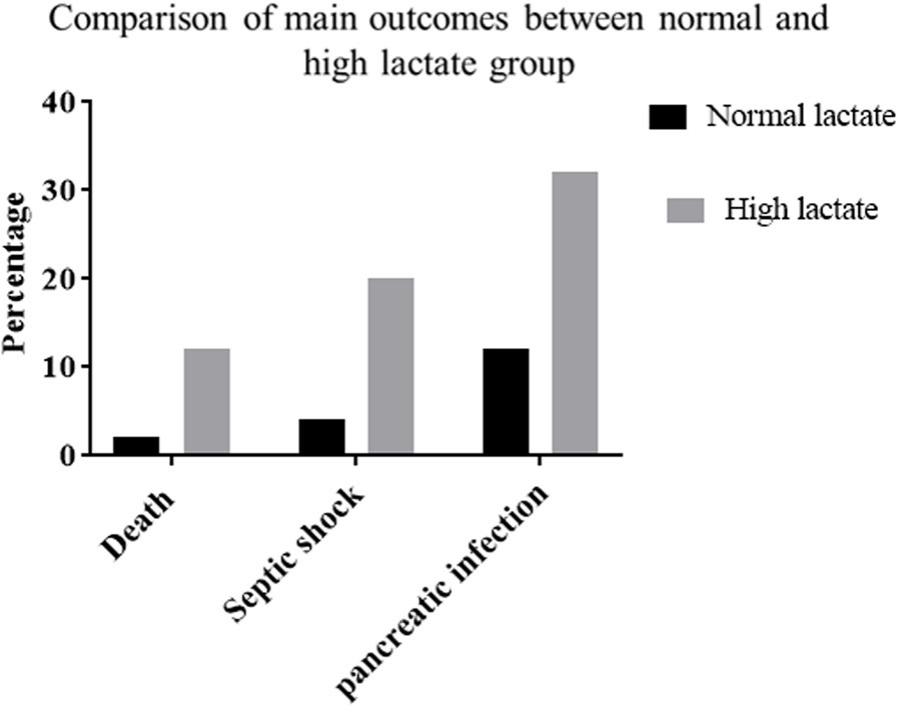
Main outcomes between normal lactate and high lactate group in severe acute pancreatitis patients

Approximately 9% of patients died in the study. For primary outcomes, we observed significant differences in death (2% vs 12%, *P* < 0.01), septic shock (4% vs 24%, *P* < 0.01) and pancreatic infection (12% vs 37%, *P* < 0.01) between the two groups (Table 3 and Fig. 2). For some of the secondary outcomes, we also observed mean differences, including abdominal compartment syndrome (3% vs 20%, *P* < 0.01), pancreatic necrosis (41% vs 63%, *P* < 0.01), walled-off necrosis (6% vs 21%, *P* < 0.01), hospital stay (13(9.5–19) vs 19 [13–30], *P* = 0.01), need for ventilator support (26% vs 54%, *P* < 0.01) and hospital stay in the ICU (6 [3–11] vs 10 [5–19], *P* < 0.01). However, for other secondary outcomes, such as portal vein thrombosis, pancreatic fistula, intestinal fistula and diabetes, there was no significant difference (Table 3).

**Table 3:** Primary and secondary outcomes according to different groups by arterial lactate

Variable	All patients (*n* = 329)	Normal (*n* = 112)	High lactate (*n* = 217)	*P* value
Primary outcomes				
Death	28 (9%)	2 (2%)	26 (12%)	0.01
Septic shock	47 (14%)	4 (4%)	43 (20%)	< 0.01
pancreatic infection	83 (25%)	13 (12%)	70 (32%)	< 0.01
Secondary outcomes				
Abdominal compartment syndrome	46 (14%)	3 (3%)	43 (20%)	< 0.01
Pancreatic necrosis	182 (55%)	46 (41%)	136 (63%)	< 0.01
Walled-off necrosis	52 (16%)	7 (6%)	45 (21%)	< 0.01
Portal vein thrombosis	19 (6%)	4 (4%)	15 (7%)	0.32
Pancreatic fistula	7 (2%)	0 (0%)	7 (3%)	0.1
Intestinal fistula	8 (2%)	0 (0%)	8 (4%)	0.06
Diabetes	51 (16%)	16 (14%)	35 (16%)	0.7
Hospital stay, days, IQR	17 (11–28)	13 (9.5–19)	19 (13–31)	0.01
Need for ventilator support	146 (44%)	29 (26%)	117 (54%)	< 0.01
Hospital stay in ICU, days, IQR	8 (4–15)	6 (3–11)	10 (5–19)	< 0.01

Data are median (IQR), or n (%); *IQR* Interquartile range, *ICU* Intensive care unit, *SIRS* Systemic inflammatory response syndrome

### Admission arterial lactate level as an independent prognostic factor for mortality

**Fig. 3:**
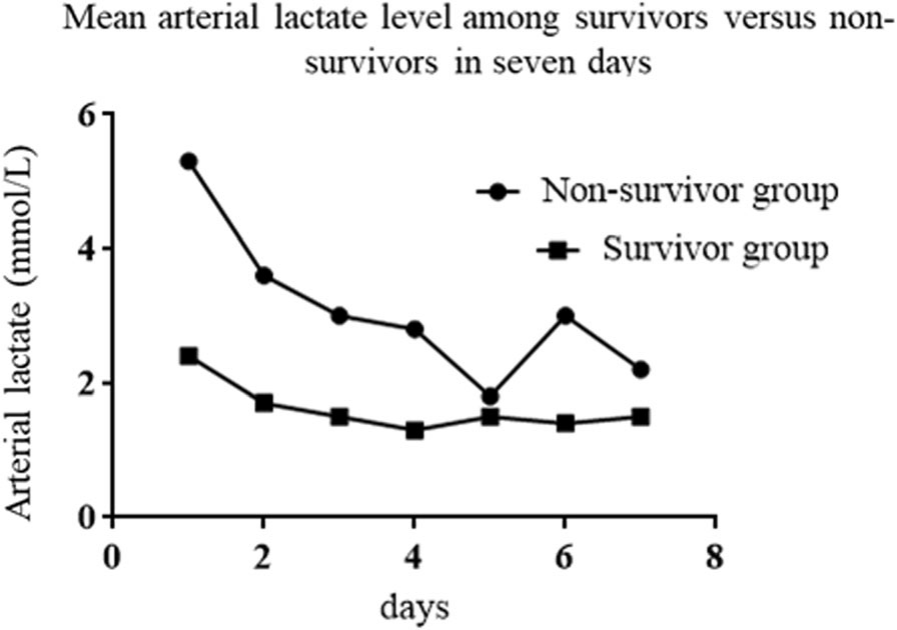
Levels and trends of arterial between survivor group and non-survivor group for severe acute pancreatitis patients in 7 days

To further evaluate the relationship between admission arterial lactate levels and death in SAP, we constructed a multivariate logistic regression analysis model consisting of five parameters (procalcitonin, arterial lactate, APACHE II, hematocrit and serum creatinine). In the multivariate logistic regression model, APACHE II, procalcitonin and arterial lactate levels were significant at *P* < 0.05, with hazard ratios 3.5(1.3–9.5), 5.6 (1.5–21.2) and 10 (3.7–27), respectively, listed in Table 4. In addition, the mean arterial lactate level in the nonsurvivor group was clearly higher than in the survivor group in the first 24 h of hospitalization (5.3 vs 2.4), and the trend in arterial lactate levels also different 7 days after enough fluid recovery (Fig. 3).

**Table 4:** Uni- and multi-variate logistic regression analyses of risk factors for mortality

Variables	Univariate analysis Hazard ratio (95%CI)	*P*-value	Multivariate analysis Hazard ratio (95%CI)	*P*-value
Procalcitonin ≥3.8 ng/ml	13.1 (3.9–44.6)	< 0.01	5.6 (1.5–21.2)	0.01
APACHEIIscore≥15	6.4 (2.9–14.3)	< 0.01	3.5 (1.3–9.5)	0.02
Arterial lactate≥4 mmol//L	14.2 (5.9–34.5)	< 0.01	10 (3.7–27)	< 0.01
Hematocrit ≥44	1.2 (0.4–1.8)	0.66	0.4 (0.2–1.3)	0.89
Serum creatinine ≥1.9 mg/dl	5.6 (2.5–12.4)	< 0.01	1.5 (0.6–4.)	0.28

Abbreviations; *APACHE* Acute Physiology and Chronic Health Evaluation

### Summary of indictors for prediction mortality in SAP

We summarized the sensitivity, specificity and AUC results in predicting mortality for arterial lactate levels as well as additional laboratory tests in Table 5. All of these parameters are moderately accurate in predicting death in SAP, with an AUC range of 0.69–0.78. For predicting mortality, an arterial lactate level ≥ 4 mmol/L had the highest accuracy, with a relatively high sensitivity of 71% and had the highest specificity of 85%, excluding the possibility of mortality. Procalcitonin ≥3.8 ng/ml had the highest sensitivity of 89% in predicting mortality.

**Table 5:** Sensitivity, specificity and AUC of different indicators in predicting mortality in SAP

Laboratory Markers	Mortality		Sensitivity (%)	Specificity (%)	AUC (%)
	Yes	No			
Arterial lactate					
≥4 mmol//L	20	45	71	85	0.78
< 4 mmol//L	8	256			
Serum creatinine					
≥1.9 mg/dl,	16	58	57	81	0.69
< 1.9 mg/ dl,	12	243			
Procalcitonin					
≥3.8 ng/ml	25	117	89	61	0.75
< 3.8 ng/ml	3	184			
APACHEII scores					
≥15	16	52	57	83	0.7
< 15	12	249			

Abbreviations; *APACHE* Acute Physiology and Chronic Health Evaluation,

## Discussion

The present study uses a medical database to investigate the association between arterial lactate levels and clinical outcomes in SAP. In this retrospective cohort study, we demonstrated that an initially elevated arterial lactate level was associated with poor outcomes and may be an effective risk-stratification biomarker in patients with SAP. Second, we found that a high arterial lactate level (≥4 mmol//L) at admission was independently associated with a risk factor for mortality after controlling for the effect of serum urea, procalcitonin, hematocrit, SIRS and APACHE II. Our study showed that an arterial lactate level ≥ 4 mmol/L on admission for the prediction of mortality in SAP had an AUC of 0.78 (95% CI: 0.68–0.88), a sensitivity of 71% and a specificity of 88% (Table 5). Furthermore, we revealed that arterial lactate levels increased again 7 days after admission after continuous fluid resuscitation in patients in the nonsurvivor group. By contrast, patients in the survivor group had normal arterial lactate levels.

Peery AE et al. reported that AP is an important cause of death and a medical burden in gastrointestinal diseases, and the overall population mortality has been unchanged in the United States [26]. Thus, AP remains a large challenge for clinicians. In 2010, a systematic review involving fourteen studies proved that organ failure and infected pancreatic necrosis were determinants of mortality in patients with AP, especially in cases of critical acute pancreatitis [25]. This implies that the ability to risk-stratify patients during the most proximal phase of SAP is of great importance for determining the need for initial intensive care and other therapeutic strategies. However, current prognostic markers in SAP have some limitations. First, the primary outcomes for most of the previously studied markers and system scores were necrosis or organ failure, rather than mortality, septic shock and pancreatic infection, which are too inconvenient to use in clinical practice to determine system scores [10, 11, 13, 15]. Inflammatory markers such as C-reactive protein and procalcitonin may be useful in predicting severity. However, it takes a great deal of time to receive the results [12]. Therefore, it is necessary to further explore new markers.

We chose the arterial lactate level as a potential marker of poor prognosis in SAP for several reasons. First, lactate is a routine laboratory marker that can both reflect the degree of tissue hypoxia and the changes in intravascular volume status. Second, as a laboratory test, a lactate measurement can be accomplished quickly with the advantages of low cost and high reproducibility. Third, as we mentioned above, in many studies, high lactate concentration portended a bad outcome in cases of critical illness, including septic shock, severe sepsis, organ failure, trauma, AP and other disease [17, 18, 22, 27]. All of those observations suggest that initially elevated arterial lactate levels may be a simple marker to risk-stratify patients with SAP in the early stages of the disease.

An important finding in our study supports the conjecture that an initially elevated arterial lactate level portends a bad outcome in SAP (Tables 2, 3). There are several possible explanations for this outcome. First, it is well known that an important reason for the worsening of AP is that insufficient blood volume, due to various causes, increases hypoperfusion of the pancreas and other vital organs, leading to pancreatic necrosis and organ dysfunction [28]. We believe that the admission arterial lactate level may reflect the underlying state of intravascular volume scarcity and vital organ perfusion (including kidney, gut, liver and even heart). Conversely, the damage to major organ functions further increases the level of arterial lactate through impaired lactate clearance and excessive production [27, 29]. Second, lactate has been regarded as a marker of tissue hypoxia in critically ill patients [21, 30]. The main function of the lung is to supply oxygen to tissues. Thus, lactate is likely to represent the degree of pulmonary failure. Persistent pulmonary failure is usually the direct cause of death in SAP. Third, lactate is also considered an early biomarker of the response to inflammatory mediators [31–33]. Lactic acid may indicate the extent of the inflammatory response in SAP. Mofidi R et al. found that persistent SIRS is associated with multiple organ dysfunction syndrome and death in patients with acute pancreatitis and is an early indicator of the likely severity of acute pancreatitis [6]. Fourth, patients with SAP have high energy metabolism [19, 34], which may be achieved by increasing aerobic glycolysis. If the amount of pyruvic acid produced by aerobic glycolysis exceeds the metabolic capacity of pyruvate dehydrogenase, it will lead to the accumulation of lactate. Levy et al. revealed that elevated lactate may be associated with increasing Na^+^ K^+^ ATPase activity in septic shock patients [21]. Therefore, lactate may also reflect the state of energy metabolism in SAP. Regardless of the specific mechanism involved, our research clearly showed that patients with elevated arterial lactate levels with early-stage SAP have an increased risk of mortality and of both mild and serious complications.

The second important finding in our research is that the average arterial lactate level in the mortality group increased again after adequate volume resuscitation (Fig. 3). This may reflect a failure of fluid resuscitation and may indicate the state of organ function, which involves lactate clearance and production. Haas SA reported that patients with a lactate clearance of less than 30% within the first 12 h had a very poor prognosis with an ICU mortality of almost 100% [18].

Our study is clearly limited in several aspects. First, this is a retrospective study, which is potentially prone to selection bias. To minimize the possibility of selection bias, we adopted strict inclusion criteria and expanded the sample size as much as possible (*N* > 300). Second, some patients received repeated measurements of lactate in 24 h based on the condition of the disease, and we took the initial value on the first day and the highest value during the following 6 days. We adopted the approach that the mean value and the lactate value on the next day must be separated by 6 h to reduce this impact. Third, some patients received rehydration therapy before admission. Finally, we followed up 1 month after the patient was discharged from the hospital, which may be too short a timeframe to adequately assess primary and secondary outcomes.

Despite these limitations, this retrospective study can provide effective information on treatment strategies. First, initially elevated admission arterial lactate levels may be a signal that patients in SAP are at risk for poorer outcomes and need to undergo aggressive resuscitation. For several decades, many clinical trials have shown that aggressive hydration may be associated with reduced morbidity and mortality [35–37]. Second, serial measurements of arterial lactate levels may offer valuable clinical information on disease progression.

## Conclusions

In summary, our research demonstrates that initially an elevated arterial lactate level is associated with morbidity and mortality in SAP. Additionally, serial monitoring of lactate levels may provide important information about disease progression. Based on the results of this study, the measurement of arterial lactate may be a useful, simple, rapid way to identify high-risk patients with SAP. Certainly, a large sample and prospective studies are needed to further confirm our results.

## Data Availability

All data generated or analyzed during this study are included in this published article [and its supplementary information files].
